# Evaluating the impact of ecological factors on the quality and habitat distribution of *Lonicera japonica* Flos using HPLC and the MaxEnt model

**DOI:** 10.3389/fpls.2024.1397939

**Published:** 2024-08-06

**Authors:** Jiali Cheng, Fengxia Guo, Liyang Wang, Zhigang Li, Chunyan Zhou, Hongyan Wang, Wei Liang, Xiaofeng Jiang, Yuan Chen, Pengbin Dong

**Affiliations:** ^1^ College of Agronomy, College of Life Science and Technology, Gansu Provincial Key Lab of Good Agricultural Production for Traditional Chinese Medicines, Gansu Provincial Engineering Research Centre for Medical Plant Cultivation and Breeding, Gansu Provincial Key Lab of Arid Land Crop Science, Gansu Key Lab of Crop Genetic and Germplasm Enhancement, Gansu Agricultural University, Lanzhou, China; ^2^ Longxi County Agricultural Technology Extension Center, Dingxi, Gansu, China; ^3^ School of Economics and Management, Hexi University, Zhangye, China; ^4^ Dryland Agriculture Institute of Plant Protection, Gansu Academy of Agricultural Sciences, Lanzhou, China

**Keywords:** *Lonicera japonica* Flos, species distribution, HPLC fingerprint, MaxEnt model, quality evaluation

## Abstract

**Introduction:**

The quality of traditional Chinese medicine is based on the content of their secondary metabolites, which vary with habitat adaptation and ecological factors. This study focuses on *Lonicera japonica Flos* (LJF), a key traditional herbal medicine, and aims to evaluate how ecological factors impact its quality.

**Methods:**

We developed a new evaluation method combining high-performance liquid chromatography (HPLC) fingerprinting technology and MaxEnt models to assess the effects of ecological factors on LJF quality. The MaxEnt model was used to predict suitable habitats for current and future scenarios, while HPLC was employed to analyze the contents of key compounds. We also used ArcGIS for spatial analysis to create a quality zoning map.

**Results:**

The analysis identified 21 common chromatographic peaks, with significant variations in the contents of Hyperoside, Rutin, Chlorogenic acid, Cynaroside, and Isochlorogenic acid A across different habitats. Key environmental variables influencing LJF distribution were identified, including temperature, precipitation, and elevation. The current suitable habitats primarily include regions south of the Yangtze River. Under future climate scenarios, suitable areas are expected to shift, with notable expansions in southern Gansu, southeastern Tibet, and southern Liaoning. The spatial distribution maps revealed that high-quality LJF is predominantly found in central and southern Hebei, northern Henan, central Shandong, central Sichuan, southern Guangdong, and Taiwan.

**Discussion:**

The study indicates that suitable growth areas can promote the accumulation of certain secondary metabolites in plants, as the accumulation of these metabolites varies. The results underscore the necessity of optimizing quality based on cultivation practices. The integration of HPLC fingerprinting technology and the MaxEnt model provides valuable insights for the conservation and cultivation of herbal resources, offering a new perspective on evaluating the impact of ecological factors on the quality of traditional Chinese medicines.

## 1 Introduction

Traditional Chinese medicine (TCM) is highly esteemed for its outstanding pharmacological effects and holistic treatment principles. Its value is evident not only in the prevention, treatment, and management of various diseases but also in the relatively low side effects and comprehensive body regulation (Wang et al., 2021; Zhang et al., 2024). The uniqueness of Chinese medicine lies in its roots in millennia of practical experience, accumulating a wealth of clinical data and forming a distinctive therapeutic system. However, the intricate composition and dynamic changes in TCM ingredients pose challenges to quality control (Xing et al., 2023). The quality of TCM is closely related to its genetic and environmental factors. Genes regulate the expression of enzymes in the metabolic pathway of active ingredients, which is the internal factor that determines the quality of TCM (Yang et al., 2022). The ecological environment is an extrinsic factor that directs effects not only on the growth, development, and distribution of plants but also on internal compositions (Neugart et al., 2018). Different environmental factors have different effects on the quality and efficacy of TCM, such as climate, soil, and altitude. Concerningly, the impacts of climate warming and more severe weather extremes, such as intensified droughts, heavy rainfall, heat waves, and cold snaps, may potentially modify the existing habitat suitability, distribution, and phenology of species, posing a threat to their survival (Aguirre-Liguori et al., 2019; Ramírez-Preciado et al., 2019). Therefore, developing effective analytical methods to study the relationship between TCM and ecological factors is of great significance for the selection and quality control of artificial cultivation areas for TCM.

*Lonicera japonica* Flos (LJF), a TCM with a significant historical usage, was initially recorded in the “MIN YI BIE LU” during the Liang Dynasty (Zheng et al., 2022a). It is also recognized as a food substance in the “Homologous Catalog of Medicine and Food (2018)” by the National Health Commission (Yang et al., 2019). Recognized for its therapeutic effects, LJF demonstrates properties such as heat-clearing, detoxification, antibacterial, and anti-inflammatory actions (Han et al., 2016; Nam et al., 2016). It serves as a pivotal treatment for ulcers and a foundational remedy for febrile diseases. Additionally, LJF can be applied in addressing various conditions, including respiratory infections and bacterial dysentery (Shang et al., 2011; Zang et al., 2022). Renowned for its exceptional antibacterial and anti-inflammatory efficacy, LJF is acclaimed as the “plant antibiotic” and has played a vital role in TCM for preventing and treating atypical pneumonia, novel coronavirus infections, and similar conditions (Li et al., 2019; Zhao et al., 2021; Yeh et al., 2022). The high medicinal value of LJF comes from its rich content of bioactive compounds, encompassing volatile oils, phenolic acids, cyclic terpenes, and flavonoids (Wang et al., 2016; Zheng et al., 2022a). Specifically, flavonoids are pivotal active components in LJF, contributing to its ability to resist bacterial and viral invasions and reduce the risk of cellular carcinogenesis (Ge et al., 2018). Key flavonoids found in LJF include rutin, hyperoside, and luteolin (Liu et al., 2020). Phenolic acid compounds, classified as organic acids, play a crucial role in the heat-clearing and detoxifying effects of LJF (Jeong et al., 2023). LJF exhibits strong adaptability, thriving in various conditions such as sunlight, shade, sandy soil, and slightly acidic to alkaline environments (Wang et al., 2023a). Its wide distribution spans from the northern regions of the three eastern provinces to the southern areas of Guangdong and Hainan Island, extending eastward to Shandong and westward to the Himalayas. Investigating whether regional differences result in variations in the quality of medicinal plants is a worthwhile endeavor.

With the advancement of chromatographic technology and computer software, the detection method has emerged as a focal point for researchers. These methods can qualitatively and quantitatively analyze the chemical components of TCM with characteristics and integrity. High-performance liquid chromatography (HPLC) stands out as a significant and contemporary separation and analysis technology, offering advantages such as efficient separation, high detection sensitivity, rapid analysis, wide applicability, and user-friendly operation, making it widely utilized in research (Yu et al., 2006; Yang et al., 2017; Arabi et al., 2018). This method encompasses the analysis of both known and unknown components, providing a comprehensive overview of the chemical composition and serving as a robust quality control approach. HPLC has become an essential tool for the quality control of Chinese medicinal materials.

The MaxEnt model, a component of the species distribution model (SDM), has been extensively employed for predicting the potential distribution range of species. This is attributed to its advantages, including high modeling accuracy, stability, and effectiveness (Phillips and Dudík, 2008; Leanza et al., 2021; Lu et al., 2024). A Geographic Information System (GIS) possesses robust spatial analysis capabilities, enabling the quantitative study of ecologically suitable habitats and providing an intuitive representation of these habitats (Leanza et al., 2021). In recent years, the integration of GIS and MaxEnt has emerged as a crucial method for assessing the impact of environmental factors on the distribution and quality of medicinal plants. It facilitates the spatial quantification of TCM quality (Zhan et al., 2022).

In recent years, researchers have utilized representative secondary metabolites and ecological factors in certain plants as indicators to assess the impact of ecological factors on plant quality and forecast suitable distribution or planting areas. For instance, Wan et al. employed the MaxEnt model and ultrahigh-performance liquid chromatography (UPLC) to assess the potential habitat suitability distribution of *Codonopsis pilosula* in Dingxi, establishing a relationship between environmental factors and active ingredient content (Wan et al., 2021). In a similar vein, Zheng et al. utilized UPLC-MS/MS to determine amide concentrations and, in conjunction with the MaxEnt model and 19 environmental factors, predicted the suitable distribution region of Red huajiao (Zheng et al., 2022b). Consequently, establishing an evaluation method for chemical composition content and ecological factors is essential for the quality assessment of TCM.

**Figure 1:**
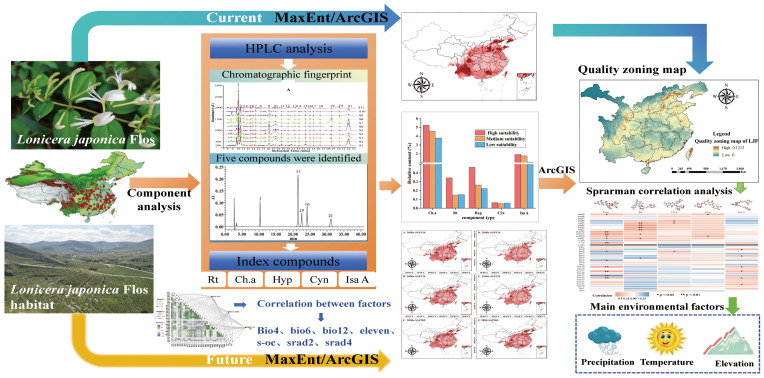
Schematic illustration of the proposed strategy for evaluating the influence of ecological factors on the quality of LJF.

Ecological factors not only affect the suitable cultivation areas of Chinese medicinal materials but also influence the formation and accumulation of chemical components in TCM. The primary objectives of this study are as follows: (1) establish a MaxEnt model to predict the distribution and delineate suitable habitats, and identify the key ecological factors influencing the distribution of LJF; (2) utilize HPLC fingerprinting technology to analyze the impact of habitat adaptability on the quality of medicinal herbs, and develop correlation models between ecological factors and medicinal efficacy components; and (3) based on the correlation models and utilizing ArcGIS software, create a quality zoning map for LJF, visually illustrating the regions where high-quality LJF is distributed. The obtained results can serve as a scientific reference for future quality control and cultivation area selection of LJF. The proposed strategy is visually represented in **Figure 1**.

## 2 Materials and methods

### 2.1 Geographic distributions of species

**Figure 2:**
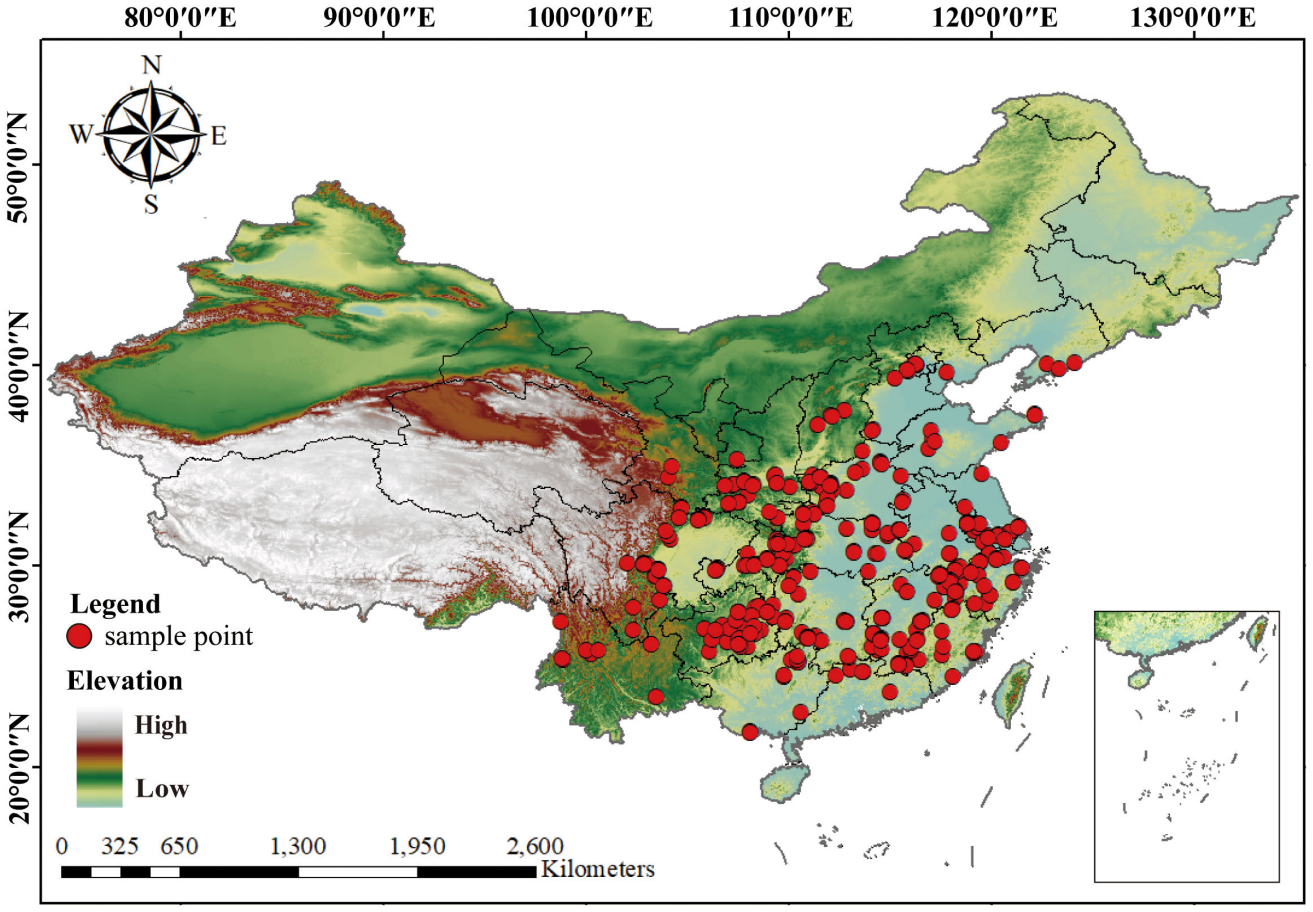
LJF included in this study, their collection sites and geographical coordinates.

Species distribution data for LJF were collected through field surveys conducted in major growing areas in China from 2023. These sample areas included Gansu, Shanxi, Sichuan, Hunan, Hubei, Hebei, Henan, Shandong, Guangdong, Jiangxi, and Yunnan. The selection of these sample sites aimed to comprehensively cover the primary LJF-producing regions. These samples were identified by Professor Chen Yuan of Gansu Agricultural University. Additionally, we complemented by resource survey reports and literature reports, as well as various online databases, including the Global Biodiversity Information Facility (GBIF, https://www.gbif.org/), the Chinese Virtual Herbarium database (CVH, http://www.cvh.ac.cn/), Flora of China, and regional flora databases, to obtain distribution points of LJF. In cases where specific geo-coordinates were lacking in certain records, Google Earth 7.0 was utilized to estimate latitude and longitude based on the described geographical locations. Subsequently, a geographic distribution map was created using ArcGIS 10.8. with map data sourced from the National Basic Geographic Information Center (http://www.ngcc.cn/ngcc/). In total, 298 specimen record points for LJF were ultimately identified and screened (**Figure 2**).

### 2.2 Environmental variables

**Figure 3:**
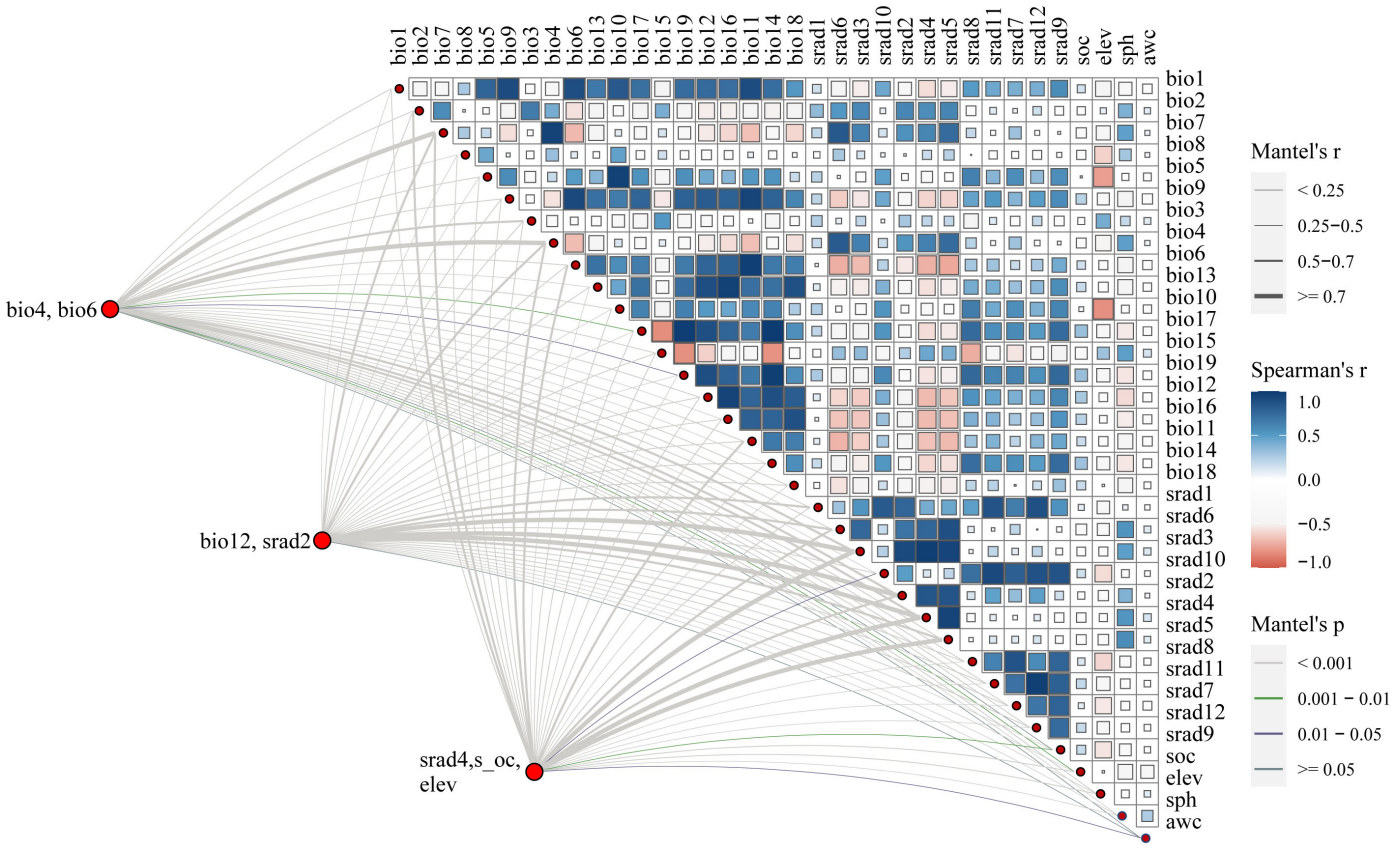
Correlation between environmental factors.

We utilized a total of 36 environmental factors, comprising 19 bioclimatic variables, 3 soil variables, elevation, awc (AWC Range), and solar radiation variables (**Table 1**). The 19 bioclimatic variables, solar radiation, and elevation data were presented in grid format with a spatial resolution of 2.5 arcmin, approximately 1 km², obtained from the WorldClim database (https://www.worldclim.org) (Fick and Hijmans, 2017). Soil data were sourced from the Harmonized World Soil Database (HWSD, http://www.fao.org/soils-portal/) (Sanchez et al., 2010). For predicting future distributions, three shared socioeconomic pathways (SSP126, SSP370, and SSP585) were downloaded (BCC-CSM2-MR), representing radiative forcing levels in 2100 (Reddy and Saravanan, 2023). SSP126 (radiation intensity of 2.6 W/m²) reflects a low-emission scenario, SSP370 (radiation intensity of 7.0 W/m²) represents a medium-emission scenario, and SSP 585 (radiation intensity of 8.5 W/m²) corresponds to a high-emission scenario (Zheng et al., 2022b). We utilized the 2050s and 2090s to predict the future potential distribution of LJF. High correlation among environmental variables can lead to overfitting of the model. To avoid high correlation among variables, a correlation analysis was performed on the initial set of 36 environmental variables. Variables with a correlation coefficient |*r*| ≥ 0.8 were considered, and among them, those with significant contribution values were selected (Dong et al., 2022; He et al., 2021a; Herrando-Moraira et al., 2019). Ultimately, seven factors were identified and retained: bio4, bio6, bio12, eleven, s-oc, srad2, and srad4 (**Figure 3**).

**Table 1:** Ecological factor variable information.

Abbreviation	Ecological factors	Unit
bio1	Annual mean temperature	°C
bio2	Mean diurnal range	°C
bio3	Isothermality	1
bio4	Temperature seasonality	1
bio5	Max temperature of warmest month	°C
bio6	Min temperature of coldest month	°C
bio7	Temperature annual range	°C
bio8	Mean temperature of wettest quarter	°C
bio9	Mean temperature of driest quarter	°C
bio10	Mean temperature of warmest quarter	°C
bio11	Mean temperature of coldest quarter	°C
bio12	Annual precipitation	mm
bio13	Precipitation of wettest month	mm
bio14	Precipitation of driest month	mm
bio15	Precipitation seasonality	mm
bio16	Precipitation of wettest quarter	mm
bio17	Precipitation of driest quarter	mm
bio18	Precipitation of warmest quarter	mm
bio19	Precipitation of coldest quarter	mm
elev	Elevation	m
s_-_ph	Subsoil pH	−log(H^+^)
s_-_cec	Subsoil CEC	cmol·kg^−1^
s_-_oc	Soil organic carbon	% weight
srad (1-12)	Solar radiation	kJ·m^−2^·day^−1^
awc	AWC range	code

### 2.3 Species distribution modeling process

The MaxEnt 3.4.1 software integrates environmental variables and distribution data for predicting species distribution and habitat (Tang et al., 2021). Prior to model establishment, the TIFF (tag image file format) format of each variable is converted to the ASC (action script communication) format. The training set is set to 75%, and the test set is set to 25% in the model (Zhang et al., 2021). We employed 100 repetitions in “Bootstrap” mode with the output format set to “Logistic,” maintaining default settings for other parameters (500 iterations, 0.00001 convergence threshold, and 10,000 background points) (Shen et al., 2022). The weights of each factor in the suitable area of LJF were assessed using the knife-cutting method, identifying key limiting factors influencing the distribution of LJF. Model accuracy was evaluated using the ROC (receiver operating characteristic curve) and the area under the curve (AUC) value. The AUC value, ranging from 0.5 to 1.0, is widely used to assess the accuracy of prediction models, with higher values indicating greater accuracy (Vanagas, 2004; Wang et al., 2019). Based on the MaxEnt model results, the suitability distribution range of LJF in China was extracted using ArcGIS software.

The suitability was divided into four grades by Jenks’ natural breaks, namely, no suitability (0–0.2), low suitability (0.2–0.4), medium suitability (0.4–0.6), and high suitability (0.6–1), to obtain the potential distribution area of LJF (Zheng et al., 2022b; Shen et al., 2022).

### 2.4 Centroid migration in the core distribution

The SDM toolbox, integrated into ArcGIS, is a software package designed to calculate changes in the suitable area for species. It helps determine the migration direction and distance of species over time. In this study, the core changes in LJF distributions were identified by converting predictions of species’ suitable distributions into binary vectors, where the species suitability probability *p* ≥ 0.04 is considered to be the total suitable region, while *p* < 0.04 is considered to be unsuitable. Then, using the Spatial Analysis Tools, the centroid coordinates of the LJF were precisely located based on different climate predictions. Finally, changes in the distribution were examined by tracking the shifts in the centroid across various SDMs

### 2.5 Preparation of sample solution

Weigh 1.0 g of LJF powder and place it in a stoppered conical flask. Add 50 mL of 50% methanol, record the mass, and perform ultrasonic extraction for 30 min. After cooling, compensate for the weight loss with 50% methanol, shake the mixture, and filter it. Then, filter the sample vial with a 0.22-μm microporous membrane and store it in the sampling bottle for HPLC analysis.

### 2.6 HPLC analysis

To establish HPLC fingerprints, the chromatographic conditions were optimized and the established method was verified. (1) Method Establishment: On the HPLC detection platform, the analytical conditions were set as follows: (a) Column, Symmetry C18 (5 µm, 4.6 mm * 250 mm); (b) Mobile phase: Acetonitrile (Merck) as mobile phase A, ultrapure water with 0.3% methanoic acid (Aladdin) as mobile phase B; (c) The gradient elution program is shown in **Table 2**; (d) Wavelength, column temperature, flow rate, and injection volume: 245 nm, 38°C, 1 mL min^−1^, and 10 μL, respectively. (2) Method Validation: Evaluate precision by injecting the same sample solution six times in a row, and assess repeatability by analyzing six different sample solutions prepared in parallel with the same procedure and batch of LJF. Stability tests of the sample solutions are conducted by analyzing them at 0-, 2-, 4-, 8-, 16-, and 24-h intervals (Jin et al., 2009; Wan et al., 2021).

**Table 2:** Gradient elution program of HPLC fingerprints.

Time (min)	Flow rate (mL min^−1^)	A%	B%
Initial	1	9	91
10	1	15	85
15	1	17	83
20	1	17.5	82.5
30	1	17.8	82.2
35	1	19	81
40	1	22	78
50	1	9	91

### 2.7 Correlation analysis between ecological factors and chemical components

For the sake of studying the effect of habitat suitability on the accumulation of ecological factors, we extracted the ecological factor values of the sampling points by ArcGIS based on the longitude and latitude information of LJF. Then, the stepwise regression method was performed using R 4.3.2 software to conduct a correlation analysis of the chemical composition of medicinal materials, including Hyperoside (Hyp), Rutin (Rt), Chlorogenic acid (Ch.a), Cynaroside (Cyn), Isochlorogenic acid A (Isa A), and ecological factors. Finally, a correlation analysis was constructed to assess the relationship between the chemical composition and ecological factors. This analysis was superimposed with the habitat suitability distribution map of LJF to create a medicinal quality zoning map.

## 3 Results and discussion

### 3.1 Accuracy of model analysis

**Figure 4:**
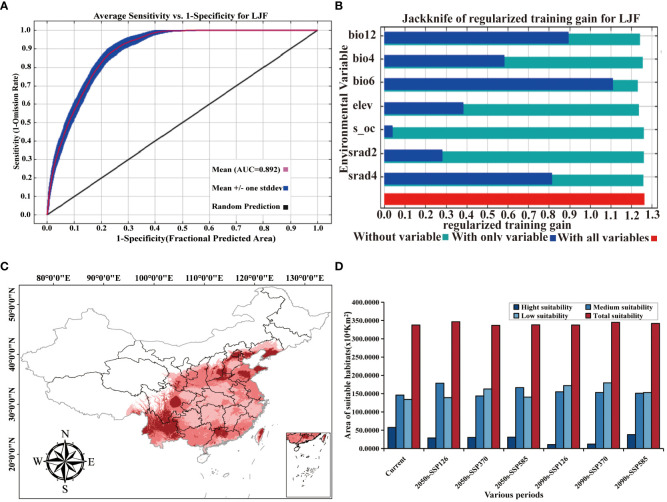
**(A)** ROC curve and AUC values. **(B)** Jackknife test results of ecological factors to LJF under modern climate conditions. **(C)** Prediction of LJF suitable area under modern climate conditions. **(D)** Suitable area (km^2^) for LJF in China under different climate conditions.

The AUC value (area under the ROC curve), being unaffected by the threshold, is widely used to assess the accuracy of prediction models (Zheng et al., 2022b). An AUC between 0.9 and 1 signifies an “excellent” model, while an AUC between 0.8 and 0.9 indicates a “good” model (Swets, 1988). In the context of ecological factors, the ROC curve generated by the MaxEnt model illustrates an AUC value of 0.892 for the LJF distribution model based on 36 environmental variables (**Figure 4A**). This value surpasses 0.8, indicating the model’s good accuracy, and the prediction results can be used for studying the division of LJF suitable areas.

### 3.2 Important environmental variables

**Figure 5:**
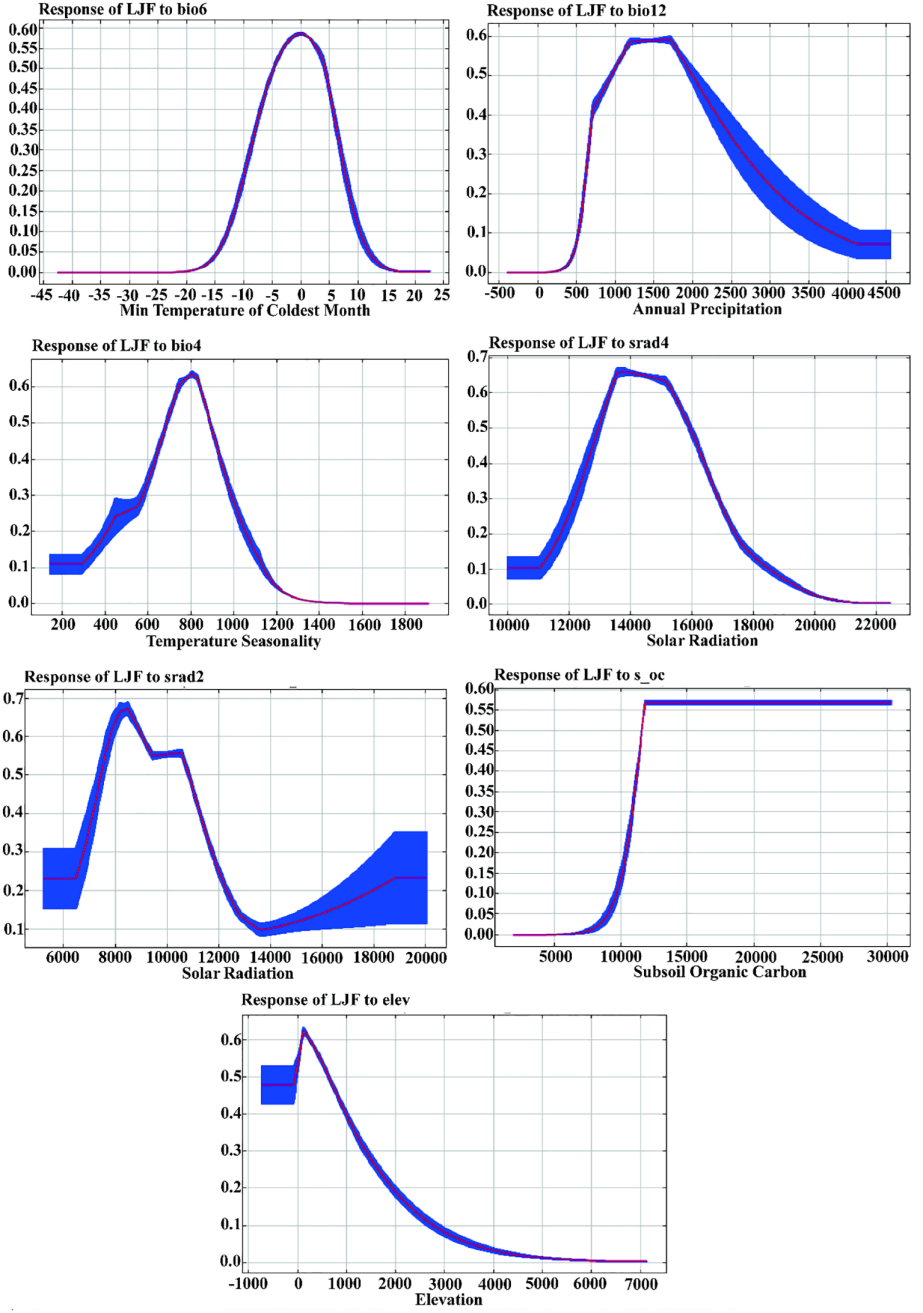
Response curves of the current existence probability of LJF to the environmental variables.

**Supplementary Table S1** provides an overview of the contribution and significance of Maxent model factor variables in shaping the distribution of LJF. In the current period, the seven variables that contribute the most to LJF are bio6, bio12, bio4, srad2, srad4, elev, and s_oc, with contribution rates of 44.6%, 40.3%, 8.5%, 3.2%, 2.4%, 0.8%, and 0.1%, respectively. The permutation importance rates for these variables are 58.1%, 25.5%, 4.6%, 0.5%, 7.7%, 2.2%, and 0.4%, respectively. Jackknife test simulations for the current period demonstrated that when simulations were conducted with a single variable, bio6 had the highest regularization training gain, indicating its provision of more effective model simulation information (**Figure 4B**). When the bio6 variable was removed from the simulation, the variable with the greatest decrease in regularization training gain was also bio6, suggesting that this variable had information about the other variables. Utilizing response curves (**Figure 5**), we determined thresholds (presence probability *p* > 0.55) for key biotic factor parameters. The identified thresholds for these parameters are as follows: Min temperature of the coldest month (bio6) ranges from −4 to 4°C, temperature seasonality (bio4) spans 700 to 900, annual precipitation (bio12) falls between 500 and 1,000 mm, solar radiation in February (srad2) varies from 7,500 to 9,000 kJ·m^−2^·day^−1^, solar radiation in April (srad4) ranges from 13,500 to 14,500 kJ·m^−2^·day^−1^, subsoil organic carbon (s_oc) at 11,000, and elevation (elev) from 0 to 2,500 m. The results showed that temperature, precipitation, elevation, and soil characteristics are the primary ecological factors influencing the growth of LJF. LJF thrives in temperatures approximately 20°C (Zheng et al., 2022a). Extreme temperature will damage the photosynthetic activity of plants, affect the stability of proteins, accumulate excessive reactive oxygen species (ROS), and change the production and signal transduction of plant hormones, thus having a significant harmful effect on plant growth, development, quality, and so on (Ohama et al., 2017; Li et al., 2018; Huang et al., 2023). LJF typically thrives in precipitation ranging from 600 to 1,000 mm. Moreover, water availability significantly impacts seedling emergence and plant growth (Khaeim et al., 2022). Long-term drought will restrict the growth and development of plants, and the photosynthesis of plant leaves will also begin to decline, and the accumulation of secondary metabolites will be weakened (Chaves et al., 2009; He et al., 2021b; Yang et al., 2007). Altitude emerges as the dominant ecological factor influencing the pharmacodynamic components of LJF. The content of components increases with the elevation of the planting area, ranging from 4 to 2,100 m (Seyis et al., 2020). LJF, being a vine or woody plant, exhibits a preference for deep soil. Research indicates that the ideal soil type for LJF is neutral or slightly alkaline, characterized by loose, deep, fertile sandy soil, and a higher soil exchange capacity (Yang et al., 2017).

### 3.3 Prediction of a suitable area for LJF under modern environmental variables

Initially predicted using the MaxEnt model, the distribution of LJF under current ecological factors is illustrated in **Figure 4C**. The identified distribution areas in northern Yunnan and Guangdong, central Sichuan and Hebei, central and eastern Shandong, and southern Liaoning align with relevant records (Sun et al., 2023). Subsequently, ArcGIS software was employed to reclassify the LJF distribution, leading to the determination of the potential suitable distribution area under the current ecological factors, as depicted in **Figure 4D**. The results indicate that the potential suitable distribution area of LJF covers 3,376,198 km^2^, constituting 35.17% of the national land area. The highly suitable habitat area covered an expanse of 574,879 km^2^, spanning the central and western parts of Sichuan (Chengdu and Ziyang), the southern part of Shandong (Linyi), the northern part of Henan (Xinxiang and Anyang), the southern and central eastern parts of Hebei (Xingtai and Chengde), the central and western parts of Guangdong (Qingyuan and Shaoguan), the southeastern part of Shanxi (Jincheng), the southern part of Liaoning (Dalian), Yunnan, and Taiwan. Moderately suitable areas are primarily distributed in the Taihang Mountains, Hengduan Mountains, Wuyi Mountains, Loess Plateau, and the middle and lower reaches of the Yangtze River, covering an area of 1,460,417 km^2^. This indicates that the modern potential distribution suitability areas simulated by MaxEnt largely align with contemporary distribution records. In summary, LJF exhibits high adaptability in the Huaihe River Basin, Qinling, and North China Plain. The warm and humid climate, along with sufficient rainfall in most areas of Sichuan, Yunnan, and Guangdong in the southwest and southern regions, creates a conducive ecological environment for the growth of LJF. These findings further emphasize LJF’s adaptability to diverse ecological conditions.

### 3.4 Potential distribution of LJF under future climate change scenarios

**Figure 6:**
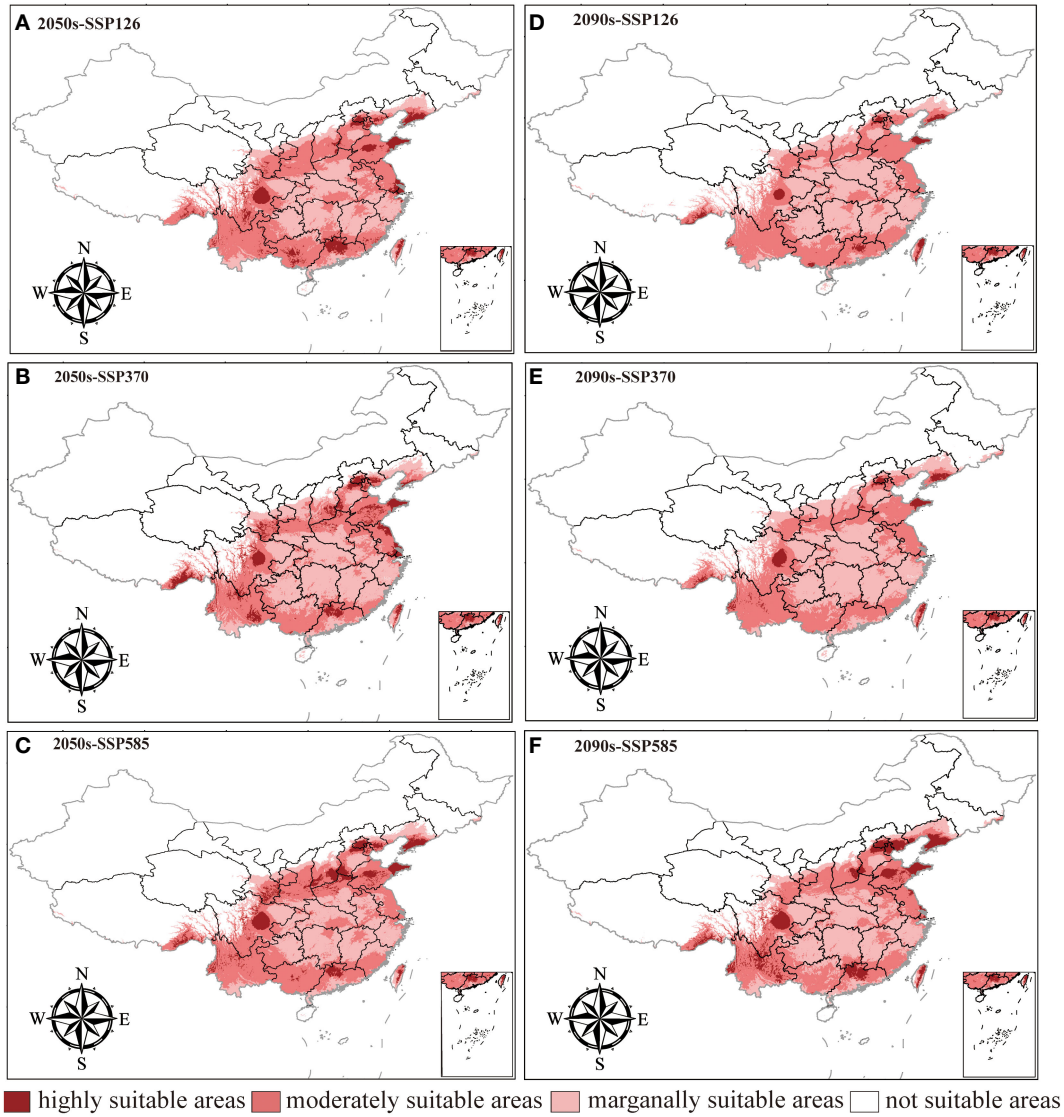
Prediction of LJF suitable area under future climate conditions. **(A)**. 2050s-SSP126, **(B)**. 2050s-SSP370, **(C)**. 2050s-SSP585, **(D)**. 2090s-SSP126, **(E)**. 2090s-SSP370, **(F)**. 2090s-SSP585.

The changes in the distribution of LJF under different future climate scenarios are depicted in **Figure 6** and **Supplementary Table S2**. In comparison to the present conditions, the total suitable area is projected to increase in the future, although the area of high suitable habitat is expected to decrease. For the period from 2041 to 2060, the highly suitable habitat areas under the SSP126, SSP370, and SSP585 climate scenarios are 288,333 km^2^, 302,170 km^2^, and 311,684 km^2^, respectively. These figures represent a reduction of 49.84%, 47.44%, and 45.78%, respectively, compared to the current climate scenarios. The moderately suitable habitat areas under these scenarios increased by 22.38%, decreased by 1.58%, and increased by 14.06%, respectively. For the period from 2081 to 2100, the highly suitable habitat area under SSP126, SSP370, and SSP585 was 106,024 km^2^, 120,035 km^2^, and 379,427 km^2^, respectively. All these values significantly decrease compared to current conditions. In contrast, the area of moderately suitable habitat increased. This is believed to be attributed to the gradual increase in greenhouse gas emissions, where elevated temperatures will limit plant growth, leading to the gradual transformation of highly suitable habitat areas into moderately suitable ones (Wan et al., 2023). In the future, LJF high suitability areas are expected to expand in southern Gansu, southeastern Tibet, and southern Liaoning (**Supplementary Figure S1**). Compared with current climate conditions and future emission scenarios, the LJF high suitability area expands to higher-altitude areas. In future emission scenarios, rising temperatures associated with increased concentrations of CO_2_ released can lead to species migration towards higher-altitude or -latitude areas (Bertrand et al., 2011; Pernicová et al., 2024).

### 3.5 Changes in gravity center of the highly suitable distribution of LJF in the future

**Figure 7:**
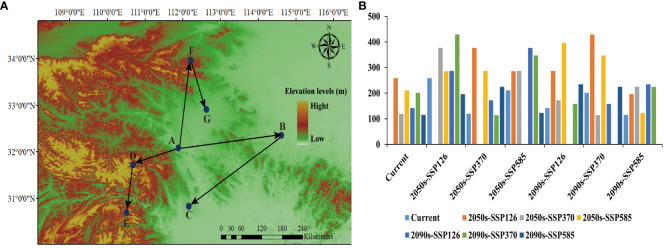
Interglacial period (**(A)** migration route; **(B)** migration distance). Among them, the meaning of the letters were (A) current, (B) 2050s-SSP126, (C) 2090s-SSP126, (D) 2050s-SSP370, (E) 2090s-SSP370, (F) 2050s-SSP585, (G) 2090s-SSP585.

The SDM_Toolbox within ArcGIS 10.8 was utilized to calculate the centroid distribution of LJF suitable areas under various environmental scenarios and time periods, thereby deriving the centroid migration trajectory (**Figure 7**). Currently, the center of gravity of the suitable habitat for LJF is in Fancheng District, Fanyang City, Hubei Province (111.881004 E, 32.083149 N). Under the 2050s-SSP126 climate scenario, the center of gravity for highly suitable areas will shift eastward to Xixian County, Xinyang City, Henan Province (114.613104 E, 32.35066 N), with a migration distance of 258.73 km. Under the 2090s-SSP126 climate scenario, the center of gravity of the suitable habitat will migrate to the Duodao District in Jingmen City, Hubei Province (112.161545 E, 30.829002 N), with a migration distance of 141.97093 km. In the scenario of 2050s-SSP370, the center of gravity of the highly suitable area will migrate westward to Shennongjia District of Hubei Province (110.682713 E, 31.722339 N), covering a migration distance of 120.02 km. In the 2090s-SSP370 scenario, the gravity center moves southwest to Changyang Tujia Autonomous County, Yichang City (110.507134 E, 30.702936 N), covering a distance of 201.38847 km. Under the 2050s-SSP585 scenario, the center of gravity of suitable habitat will migrate eastward to Songxian County, Luoyang City, Henan Province (110.682713 E, 31.722339 N), with a migration distance of 210.94 km. Under the 2090s-SSP585 scenario, the center of gravity of suitable habitat moves to Yuancheng District, Nanyang City, Henan Province (112.624916 E, 32.90641 N). Simulation of future distributions indicates that under three different climate scenarios (SSP126, SSP370, and SSP585), the suitable distribution areas for LJF are decreasing. This phenomenon is also observed in other plants such as *Leonurus japonicus* and *Leucanthemum vulgare* (Wang et al., 2023b; Ahmad et al., 2019). In the high emissions scenario for 2050, temperatures are projected to rise by approximately 1.3°C and by over 2.79°C in the 2090s (Ford et al., 2012; Brouziyne et al., 2021). Consequently, the population of LJF is expected to generally decline, and habitat fragmentation is anticipated to become more severe than it is currently. Under the emission scenarios for the 2050s and 2090s, the predicted suitable areas are projected to shift towards southern regions at higher elevations. Studies have shown that the altitudinal distribution of species is largely driven by temperature gradients (Lenoir et al., 2008; Ashraf et al., 2016; Kumar et al., 2022). However, besides the impact of temperature changes, the suitable distribution of species may also involve biological characteristics, geological factors, or other disturbance factors (Dong et al., 2022). LJF thrives in moist, warm climates. Therefore, there is a tendency to migrate to the south.

### 3.6 Chromatographic fingerprint analysis

**Figure 8:**
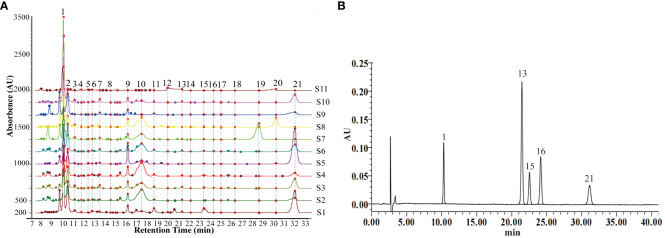
**(A)** Standard fingerprints of LJF in eleven regions. **(B)** Reference fingerprint. The serial numbers S1-11 represent Guangdong, Shandong, Yunnan, Hubei, Henan, Jiangxi, Hebei, Sichuan, Shanxi, Hunan and Gansu province, respectively. The numbers 1, 13, 15, 16, 21 represent Ch.a, Rt, Hyp, Cyn, Isa A.

Chromatographic fingerprinting enables the qualitative and quantitative assessment of TCM quality by analyzing chemical information and depicting the peak distribution of specific components. At a detection wavelength of 245 nm, there is high separation between chromatographic peaks, elevated response, symmetrical peak shapes, and a stable baseline, facilitating fingerprint establishment. The results of method validation showed that the relative standard deviation (RSD) for the precision of Rt, Ch.a, Hyp, Cyn, and Isa A were 2.3%, 2.2%, 3.6%, 3.5%, and 1.9%, respectively. The RSD for reproducibility were 2.0%, 3.7%, 3.2%, 3.1%, and 2.7%, respectively, and the RSD for stability were 3.0%, 3.2%, 2.8%, 3.1%, and 2.5%, respectively. These results indicate that the established method is suitable for the development of HPLC fingerprints of LJF. The Chromatographic Fingerprint is imported into the Similarity Evaluation System for Chromatographic Fingerprint of TCM (version 2012). Subsequently, the reference spectrum and time width are set, peak areas are normalized, multi-point correction is conducted, peaks are automatically matched, and the standard fingerprint is generated. The standard fingerprints of LJF in 11 different regions are presented in **Figure 8A**. Based on chromatographic peak matching information, 21 common peaks were selected. The similarity values ranged between 0.817 and 0.989, indicating that the chemical composition of LJF from different locations varied to some extent (**Table 3**). Five compounds, Ch.a, Rt, Hyp, Cyn, and Isa A, were identified through the control (**Figure 8B**). Subsequently, a quantitative analysis of the five components in LJF from different origins was conducted (**Supplementary Figure S2**). The results revealed that the average contents of Ch.a, Hyp, and Isa A were higher in medium and high suitable areas than in low suitable areas. The contents gradually decreased from high suitable areas to low suitable areas, whereas the contents of Rt and Cyn in low suitable areas were higher than those in medium suitable areas. This indicates that the suitable growth area is not necessarily conducive to the accumulation of all secondary metabolites. Research indicates that certain species, under the influence of environmental stress, produce specific secondary metabolites to enhance adaptability to adversity and facilitate the formation of authentic medicinal materials (Li et al., 2020).

**Table 3:** The similarity values of LJF in 11 regions.

No.	S1	S2	S3	S4	S5	S6	S7	S8	S9	S10	S11	C
S1	1											
S2	0.583	1										
S3	0.915	0.806	1									
S4	0.543	0.993	0.756	1								
S5	0.861	0.395	0.647	0.387	1							
S6	0.827	0.917	0.951	0.900	0.590	1						
S7	0.854	0.644	0.919	0.56	0.622	0.772	1					
S8	0.800	0.846	0.950	0.783	0.562	0.892	0.948	1				
S9	0.675	0.553	0.671	0.579	0.450	0.766	0.355	0.439	1			
S10	0.909	0.659	0.97	0.588	0.611	0.852	0.958	0.922	0.578	1		
S11	0.767	0.503	0.847	0.406	0.472	0.658	0.955	0.866	0.293	0.935	1	
C	0.926	0.826	0.989	0.781	0.724	0.949	0.919	0.957	0.63	0.943	0.817	1

### 3.7 Correlation analysis between ecological factors and chemical components

**Figure 9:**
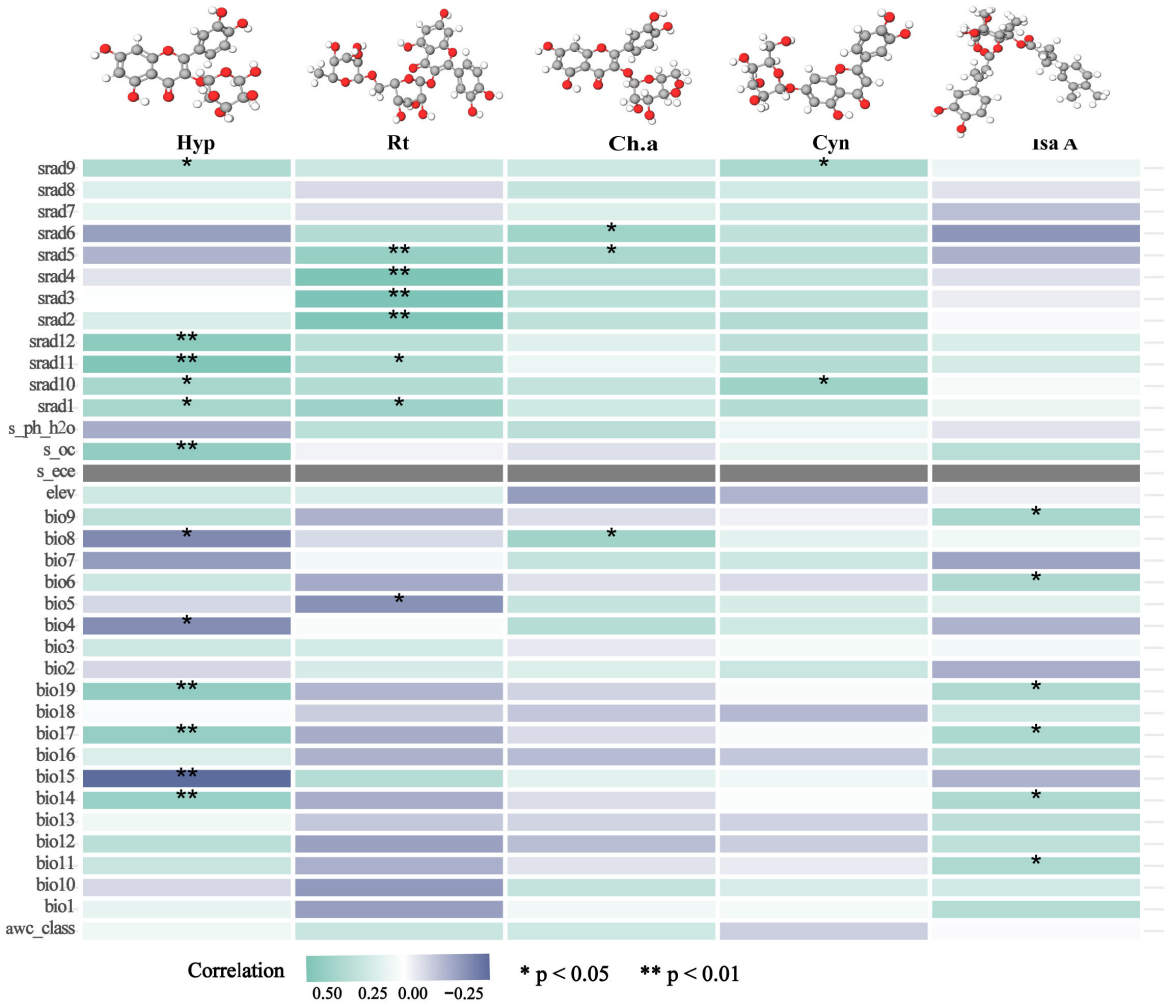
Correlation heatmap between chemical composition and ecological factors.

**Figure 10:**
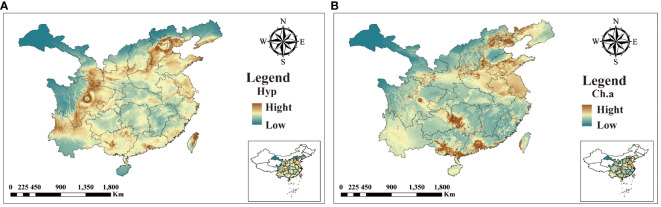
The spatial distribution of the content of Hyp **(A)** and Ch.a **(B)**.

**Figure 11:**
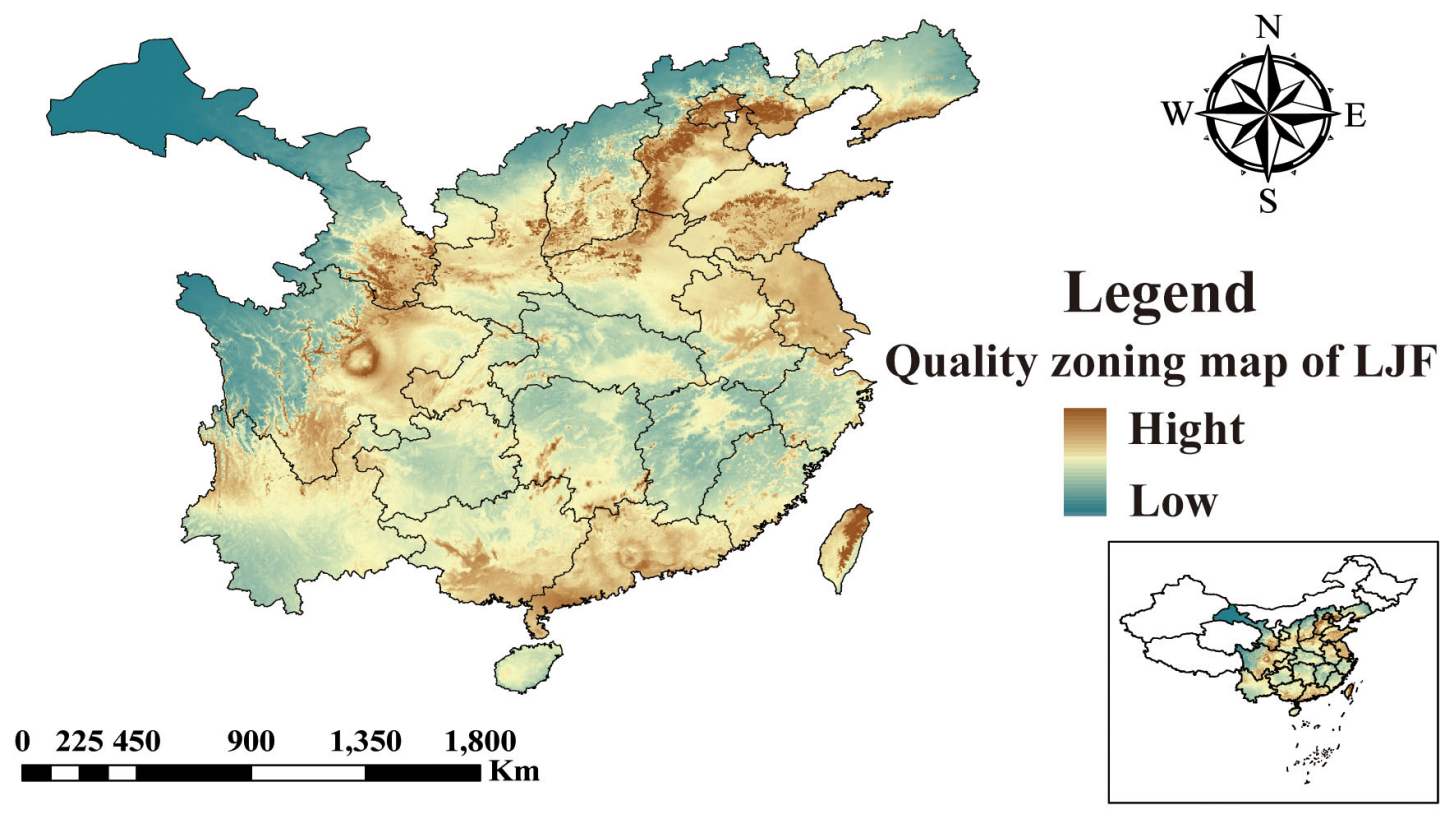
The quality zoning map of LJF in Distribution areas.

The five identified components were correlated with the ecological factors identified by the MaxEnt model (**Figure 9**), indicating a significant impact of ecological factors on these components. In LJF, Hyp and Isa A show negative correlations with srad3, srad4, srad5, and srad6, while Rt, Ch.a, and Cyn exhibit a positive correlation (*p* < 0.01). Additionally, Rt, Ch.a, and Cyn display negative correlations with bio9, bio19, bio18, bio17, bio16, bio11, bio12, bio13, and bio14, whereas they are positively correlated with bio8. Furthermore, bio8 shows a negative correlation with Hyp (*p* < 0.05) and a positive correlation with Ch.a (*p* < 0.05). The correlation between the same ecological factor and different components varies, as does the correlation between different ecological factors and the same component. To address this issue, the spatial analysis function and correlation model of ArcGIS software were employed to create spatial distribution maps for the content of four common peaks (the analysis was not conducted for the correlation between Cyn and factors due to its lack of obvious correlation). **Figure 10** illustrates the spatial distribution of the contents of Hyp and Ch.a. Ultimately, the content spatial distribution maps of LJF and the habitat suitability map were superimposed to generate the quality zoning map of LJF in the distribution area, as depicted in **Figure 11**. In **Figure 11**, high-quality LJF is predominantly distributed in central and southern Hebei Province, northern Henan Province, central Shandong Province, central Sichuan Province, southern Guangdong Province, and the Taiwan Peninsula. Poor-quality LJF is distributed in northern Gansu, western Sichuan, northeastern Liaoning, and northwestern Shanxi. By comparing the zoning map with the habitat suitability distribution map, it is evident that the low-quality area is not a suitable area. This implies that ecological factors unfavorable to the growth of LJF also hinder the accumulation of LJF pharmacodynamic components. However, suitable areas are not necessarily high-quality areas. Optimal conditions for the growth and development of certain herbs and the accumulation of secondary metabolites do not necessarily align. This discrepancy can be attributed to the complexity involved in the synthesis and accumulation of secondary metabolites in medicinal plants. Research indicates that active ingredients (secondary metabolites) are produced and accumulate under stressful (adverse) conditions. Since the active substances in medicinal plants are predominantly secondary metabolites, this is largely considered a result of plant adaptation to adversity (Al-Khayri et al., 2023). In this study, by considering the spatial correlation between habitat suitability and quality suitability, an LJF quality zoning map was obtained, facilitating LJF production zoning.

## 4 Conclusion

In this study, we established a new method to evaluate the effects of ecological factors on LJF quality based on HPLC fingerprinting technology, ArcGIS, and MaxEnt models, combined with database dates, field sampling, and component analysis. Using the MaxEnt model, the ecological factors affecting the suitable distribution area of LJF were screened as bio4, bio6, bio12, srad2, srad4, s_oc, and elev. The distribution area was divided into highly suitable, medium suitable, low suitable, and unsuitable areas. Under future climate scenarios, the high suitability zone of LJF will expand in southern Gansu, southeastern Tibet, and southern Liaoning. The correlation model between ecological factors and chemical components was established through bivariate correlation analysis. Finally, based on the correlation and ArcGIS spatial distribution model, the LJF quality distribution map is drawn to visually show the distribution area of LJF. The screened ecological factors affecting the distribution of LJF habitat adaptation were compatible with the growth habit of LJF, which verified the reliability of the method. The combination of chromatographic fingerprinting technique and MaxEnt model provides a reference for the conservation of Chinese herbal resources and the selection of cultivation areas, and also provides a new perspective for evaluating the influence of ecological factors on the quality of Chinese herbal medicines.

## Data availability statement

The original contributions presented in the study are included in the article/**Supplementary Material**. Further inquiries can be directed to the corresponding authors.

## Author contributions

JC: Investigation, Methodology, Formal analysis, Conceptualization, Data curation, Writing – original draft. FG: Methodology, Data curation, Writing – review & editing. LW: Investigation, Writing – review & editing. ZL: Formal analysis, Writing – review & editing. CZ: Formal analysis, Writing – review & editing. HW: Data curation, Writing – review & editing. WL: Investigation, Writing – review & editing. XJ: Supervision, Writing – review & editing. YC: Methodology, Writing – review & editing, Investigation, Supervision. PD: Supervision, Writing – original draft.

## References

[B1] Aguirre-Liguori J. A. Ramírez-Barahona S. Tiffin P. Eguiarte L. E. (2019). Climate change is predicted to disrupt patterns of local adaptation in wild and cultivated maize. Proc. R. Soc. B: Biol. Sci. 286, 0486. doi: 10.1098/rspb.2019.0486 PMC6650710 31290364

[B2] Ahmad R. Khuroo A. A. Charles B. Hamid M. Rashid I. Aravind N. A. (2019). Global distribution modelling, invasion risk assessment and niche dynamics of *Leucanthemum vulgare* (Oxeye Daisy) under climate change. Sci. Rep. 9, 115. doi: 10.1038/s41598-019-47859 31388050 PMC6684661

[B3] Al-Khayri J. M. Rashmi R. Toppo V. Chole P. B. Banadka A. Sudheer W. N.. (2023). Plant secondary metabolites: the weapons for biotic stress management. Metabolites 13, 716. doi: 10.3390/metabo13060716 37367873 PMC10302943

[B4] Arabi M. Ostovan A. Asfaram A. Ghaedi M. (2018). Development of an eco-friendly approach based on dispersive liquid–liquid microextraction for the quantitative determination of quercetin in *Nasturtium officinale*, *Apium graveolens*, *Spinacia oleracea*, *Brassica oleracea* var. *sabellica*, and food samples. New J. Chem. 42, 14340–14348. doi: 10.1039/C8NJ02485E

[B5] Ashraf U. Ali H. Chaudry M. N. Ashraf I. Batool A. Saqib Z. (2016). Predicting the Potential Distribution of *Olea ferruginea* in Pakistan incorporating Climate Change by Using Maxent Model. Sustainability 8, 722. doi: 10.3390/su8080722

[B6] Bertrand R. Lenoir J. Piedallu C. Riofrío-Dillon G. De Ruffray P. Vidal C.. (2011). Changes in plant community composition lag behind climate warming in lowland forests. Nature 479, 517–520. doi: 10.1038/nature10548 22012261

[B7] Brouziyne Y. De Girolamo A. M. Aboubdillah A. Benaabidate L. Bouchaou L. Chehbouni A. (2021). Modeling alterations in flow regimes under changing climate in a Mediterranean watershed: An analysis of ecologically-relevant hydrological indicators. Ecol. Inf. 61, 101219. doi: 10.1016/j.ecoinf.2021.101219

[B8] Chaves M. M. Flexas J. Pinheiro C. (2009). Photosynthesis under drought and salt stress: regulation mechanisms from whole plant to cell. Ann. Bot. 103, 551–560. doi: 10.1093/aob/mcn125 18662937 PMC2707345

[B9] Dong P. B. Wang L. Y. Wang L. J. Jia Y. Li Z. H. Bai G.. (2022). Distributional response of the rare and endangered tree species *abies chensiensis* to climate change in East Asia. Biology 11, 1659. doi: 10.3390/biology11111659 36421374 PMC9687575

[B10] Fick S. E. Hijmans R. J. (2017). WorldClim 2: new 1-km spatial resolution climate surfaces for global land areas. Int. J. Climatol. 37, 4302–4315. doi: 10.1002/joc.5086

[B11] Ford J. D. Vanderbilt W. Berrang-Ford L. (2012). Authorship in IPCC AR5 and its implications for content: climate change and indigenous populations in WGII. Clim. Change 113, 201–213. doi: 10.1007/s10584-011-0350-z 26005230 PMC4439732

[B12] Ge L. Li J. Wan H. Zhang K. Wu W. Zou X.. (2018). Novel flavonoids from *Lonicera japonica* flower buds and validation of their anti-hepatoma and hepatoprotective activity in *vitro* studies. Ind. Crops Prod. 125, 114–122. doi: 10.1016/j.indcrop.2018.08.073

[B13] Han M. H. Lee W. S. Nagappan A. Hong S. H. Jung J. H. Park C.. (2016). Flavonoids isolated from flowers of *lonicera japonica* thunb. Inhibit inflammatory responses in BV2 microglial cells by suppressing TNF-α and IL-β Through PI3K/akt/NF-kb signaling pathways. Phytother. Res. 30, 1824–1832. doi: 10.1002/ptr.5688 27534446

[B14] He P. Li J. Li Y. Xu N. Gao Y. Guo L.. (2021a). Habitat protection and planning for three Ephedra using the MaxEnt and Marxan models. Ecol. Indic. 133, 108399. doi: 10.1016/j.ecolind.2021.108399

[B15] He W. Yan K. Zhang Y. Bian L. Mei H. Han G. (2021b). Contrasting photosynthesis, photoinhibition and oxidative damage in honeysuckle (*Lonicera japonica* Thunb.) under iso-osmotic salt and drought stresses. Environ. Exp. Bot. 182, 104313. doi: 10.1016/j.envexpbot.2020.104313

[B16] Herrando-Moraira S. Nualart N. Herrando-Moraira A. Chung M. Y. Chung M. G. López-Pujol J. (2019). Climatic niche characteristics of native and invasive *Lilium lancifolium* . Sci. Rep. 9, 14334. doi: 10.1038/s41598-019-50762-4 31586099 PMC6778149

[B17] Huang J. Zhao X. Bürger M. Chory J. Wang X. (2023). The role of ethylene in plant temperature stress response. Trends Plant Sci. 28, 808–824. doi: 10.1016/j.tplants.2023.03.001 37055243

[B18] Jeong S. H. Park M. Y. Bhosale P. B. Abusaliya A. Won C. K. Park K. I.. (2023). Potential antioxidant and anti-inflammatory effects of lonicera japonica and *citri reticulatae pericarpium* polyphenolic extract (LCPE). Antioxidants 12, 1582. doi: 10.3390/antiox12081582 37627577 PMC10451293

[B19] Jin Y. Liang T. Fu Q. Xiao Y. S. Feng J. T. Ke Y. X.. (2009). Fingerprint analysis of *Ligusticum chuanxiong* using hydrophilic interaction chromatography and reversed-phase liquid chromatography. J. Chromatogr. A 1216, 2136–2141. doi: 10.1016/j.chroma.2008.04.010 18440542

[B20] Khaeim H. Kende Z. Jolánkai M. Kovács G. P. Gyuricza C. Tarnawa Á. (2022). Impact of temperature and water on seed germination and seedling growth of maize (*Zea mays* L.). Agronomy 12, 397. doi: 10.3390/agronomy12020397

[B21] Kumar D. Pandey A. Rawat S. Joshi M. Bajpai R. Upreti D. K.. (2022). Predicting the distributional range shifts of *Rhizocarpon geographicum* (L.) DC. @ in Indian Himalayan Region under future climate scenarios. Environ. Sci. pollut. Res. 29, 61579–61593. doi: 10.1007/s11356-021-15624-5 34351582

[B22] Leanza P. M. Valenti F. D’Urso P. R. Arcidiacono C. (2021). A combined MaxEnt and GIS-based methodology to estimate cactus pear biomass distribution: application to an area of southern Italy. Biofuels Bioprod. Bioref. 16, 54–67. doi: 10.1002/bbb.2304

[B23] Lenoir J. Gegout J. C. Marquet P. A. DE Ruffray P. Brisse H. (2008). A signifificant upward shift in plant species optimum elevation during the 20^th^ century. Science 320, 1768–1771. doi: 10.1126/science.1156831 18583610

[B24] Li B. Gao K. Ren H. Tang W. (2018). Molecular mechanisms governing plant responses to high temperatures. J. Integr. Plant Biol. 60, 757–779. doi: 10.1111/jipb.12701 30030890

[B25] Li Y. Kong D. Fu Y. Sussman M. R. Wu H. (2020). The effect of developmental and environmental factors on secondary metabolites in medicinal plants. Plant Physiol. Biochem. 148, 80–89. doi: 10.1016/j.plaphy.2020.01.006 31951944

[B26] Li Y. Li W. Fu C. Song Y. Fu Q. (2019). *Lonicerae japonicae* flos and *Lonicerae* flos: a systematic review of ethnopharmacology, phytochemistry and pharmacology. Phytochem. Rev. 19, 1–61. doi: 10.1007/s11101-019-09655-7 32206048 PMC7088551

[B27] Liu D. Yu X. Sun H. Zhang W. Liu G. Zhu L. (2020). Flos lonicerae flavonoids attenuate experimental ulcerative colitis in rats via suppression of NF-κB signaling pathway. Naunyn-Schmiedeberg’s Arch. Pharmacol. 393, 2481–2494. doi: 10.1007/s00210-020-01814-4 32125461

[B28] Lu W. X. Wang Z. Z. Hu X. Y. Rao G. Y. (2024). Incorporating eco-evolutionary information into species distribution models provides comprehensive predictions of species range shifts under climate change. Sci. Total Environ. 912, 169501. doi: 10.1016/j.scitotenv.2023.169501 38145682

[B29] Nam Y. Lee J. M. Wang Y. Ha H. S. Sohn U. D. (2016). The effect of Flos *Lonicerae Japonicae* extract on gastro-intestinal motility function. J. Ethnopharmacol. 179, 280–290. doi: 10.1016/j.jep.2015.12.056 26743226

[B30] Neugart S. Baldermann S. Hanschen F. S. Klopsch R. Wiesner-Reinhold M. Schreiner M. (2018). The intrinsic quality of brassicaceous vegetables: How secondary plant metabolites are affected by genetic, environmental, and agronomic factors. Sci. Hortic. 233, 460–478. doi: 10.1016/j.scienta.2017.12.038

[B31] Ohama N. Sato H. Shinozaki K. Yamaguchi-Shinozaki K. (2017). Transcriptional regulatory network of plant heat stress response. Trends Plant Sci. 22, 53–65. doi: 10.1016/j.tplants.2016.08.015 27666516

[B32] Pernicová N. Urban O. Čáslavský J. Kolář T. Rybníček M. Sochová I.. (2024). Impacts of elevated CO_2_ levels and temperature on photosynthesis and stomatal closure along an altitudinal gradient are counteracted by the rising atmospheric vapor pressure deficit. Sci. Total Environ. 921, 171173. doi: 10.1016/j.scitotenv.2024.171173 38401718

[B33] Phillips S. J. Dudík M. (2008). Modeling of species distributions with Maxent: new extensions and a comprehensive evaluation. Ecography 31, 161–175. doi: 10.1111/j.0906-7590.2008.5203.x

[B34] Ramírez-Preciado R. P. Gasca-Pineda J. Arteaga M. C. (2019). Effects of global warming on the potential distribution ranges of six *Quercus* species (Fagaceae). Flora 251, 32–38. doi: 10.1016/j.flora.2018.12.006

[B35] Reddy N. M. Saravanan S. (2023). Extreme precipitation indices over India using CMIP6: a special emphasis on the SSP585 scenario. Environ. Sci. pollut. Res. 30, 47119–47143. doi: 10.1007/s11356-023-25649-7 36732454

[B36] Sanchez A. C. Osborne P. E. Haq N. (2010). Identifying the global potential for baobab tree cultivation using ecological niche modelling. Agroforestry Syst. 80, 191–201. doi: 10.1007/s10457-010-9282-2

[B37] Seyis F. Yurteri E. Özcan A. Cirak C. (2020). Altitudinal impacts on chemical content and composition of Hypericum perforatum, a prominent medicinal herb. South Afr. J. Bot. 135, 391–403. doi: 10.1016/j.sajb.2020.09.034

[B38] Shang X. Pan H. Li M. Miao X. Ding H. (2011). *Lonicera japonica* Thunb.: Ethnopharmacology, phytochemistry and pharmacology of an important traditional Chinese medicine. J. Ethnopharmacol. 138, 1–21. doi: 10.1016/j.jep.2011.08.016 21864666 PMC7127058

[B39] Shen Y. Tu Z. Zhang Y. Zhong W. Xia H. Hao Z.. (2022). Predicting the impact of climate change on the distribution of two relict *Liriodendron* species by coupling the MaxEnt model and actual physiological indicators in relation to stress tolerance. J. Environ. Manage. 322, 116024. doi: 10.1016/j.jenvman.2022.116024 36055092

[B40] Sun Q. H. Morales-Briones D. F. Wang H. X. Landis J. B. Wen J. Wang H. F. (2023). Target sequence capture data shed light on the deeper evolutionary relationships of subgenus *Chamaecerasus* in *Lonicera* (Caprifoliaceae). Mol. Phylogenet. Evol. 184, 116024. doi: 10.1016/j.ympev.2023.107808 37156329

[B41] Swets J. A. (1988). Measuring the accuracy of diagnostic systems. Sci. Total Environ. 240, 1285–1293. doi: 10.1126/science.3287615 3287615

[B42] Tang X. Yuan Y. Li X. Zhang J. (2021). Maximum entropy modeling to predict the impact of climate change on pine wilt disease in China. Front. Plant Sci. 12. doi: 10.3389/fpls.2021.652500 PMC8102737 33968109

[B43] Vanagas G. (2004). Receiver operating characteristic curves and comparison of cardiac surgery risk stratification systems. Interact. Cardiovasc. Thorac. Surg. 3, 319–322. doi: 10.1016/j.icvts.2004.01.008 17670248

[B44] Wan G. Z. Guo Z. H. Xi S. Y. Jin L. Chen J. (2023). Spatial variability and climate response characteristics of chemical components of *Tussilago farfara* L. Ind. Crops Prod. 204, 117352. doi: 10.1016/j.indcrop.2023.117352

[B45] Wan G. Z. Wang L. Jin L. Chen J. (2021). Evaluation of environmental factors affecting the quality of *Codonopsis pilosula* based on chromatographic fingerprint and MaxEnt model. Ind. Crops Prod. 170, 113783. doi: 10.1016/j.indcrop.2021.113783

[B46] Wang K. Chen Q. Shao Y. Yin S. Liu C. Liu Y.. (2021). Anticancer activities of TCM and their active components against tumor metastasis. Biomed. Pharmacother. 133, 111044. doi: 10.1016/j.biopha.2020.111044 33378952

[B47] Wang L. Jiang Q. Hu J. Zhang Y. Li J. (2016). Research Progress on Chemical Constituents of *Lonicerae japonicae* flos. BioMed. Res. Int. 2016, 1–18. doi: 10.1155/2016/8968940 PMC4923575 27403439

[B48] Wang X. Ma B. Liu H. Bao Y. Li M. McLaughlin N. B.. (2023a). Improvement in gravel-mulched land soil nutrient and bacterial community diversity with Lonicera japonica. Front. Microbiol. 14. doi: 10.3389/fmicb.2023.1225503 PMC10733477 38130947

[B49] Wang Y. Xie L. Zhou X. Chen R. Zhao G. Zhang F. (2023b). Prediction of the potentially suitable areas of *Leonurus japonicus* in China based on future climate change using the optimized MaxEnt model. Ecol. Evol. 13, e10597. doi: 10.1002/ece3.10597 37869439 PMC10585429

[B50] Wang Y. Zhang L. Du Z. Pei J. Huang L. (2019). Chemical diversity and prediction of potential cultivation areas of cistanche herbs. Sci. Rep. 9, 19737. doi: 10.1038/s41598-019-56379-x 31875048 PMC6930302

[B51] Xing X. Sun M. Guo Z. Zhao Y. Cai Y. Zhou P.. (2023). Functional annotation map of natural compounds in traditional Chinese medicines library: TCMs with myocardial protection as a case. Acta Pharm. Sin. B 13, 3802–3816. doi: 10.1016/j.apsb.2023.06.002 37719385 PMC10502289

[B52] Yang X. Liang Z. Wen X. Lu C. (2007). Genetic engineering of the biosynthesis of glycinebetaine leads to increased tolerance of photosynthesis to salt stress in transgenic tobacco plants. Plant Mol. Biol. 66, 73–86. doi: 10.1007/s11103-007-9253-9 17975734

[B53] Yang X. Liu Y. Hou A. Yang Y. Tian X. He L. (2017). Systematic review for geo-authentic *Lonicerae Japonicae* Flos. Front. Med. 11, 203–213. doi: 10.1007/s11684-017-0504-0 28425044 PMC7089257

[B54] Yang L. Miao L. Gong Q. Guo J. (2022). Advances in studies on transcription factors in regulation of secondary metabolites in Chinese medicinal plants. Plant Cell Tissue Organ Cult. 151, 1–9. doi: 10.1007/s11240-022-02334-0

[B55] Yang B. Zhong Z. Wang T. Ou Y. Tian J. Komatsu S.. (2019). Integrative omics of *Lonicera japonica* Thunb. Flower development unravels molecular changes regulating secondary metabolites. J. Proteomics 208, 103470. doi: 10.1016/j.jprot.2019.103470 31374363 PMC7102679

[B56] Yeh Y. C. Doan L. H. Huang Z. Y. Chu L. W. Shi T. H. Lee Y. R.. (2022). Honeysuckle (*Lonicera japonica*) and Huangqi (*Astragalus membranaceus*) Suppress SARS-CoV-2 Entry and COVID-19 Related Cytokine Storm in Vitro. Front. Pharmacol. 12. doi: 10.3389/fphar.2021.765553 PMC8990830 35401158

[B57] Yu K. Little D. Plumb R. Smith B. (2006). High-throughput quantification for a drug mixture in rat plasma – a comparison of Ultra Performance™ liquid chromatography/tandem mass spectrometry with high-performance liquid chromatography/tandem mass spectrometry. Rapid Commun. Mass Spectrom. 20, 544–552. doi: 10.1002/rcm.2336 16419023

[B58] Zang Z. Zhao S. Yang M. Yu C. Ouyang H. Chen L.. (2022). Blood chemical components analysis of honeysuckle and formulation of xanthan gum/starch-based (PVA-co-AA) hydrogels for controlled release. Arab. J. Chem. 15, 104312. doi: 10.1016/j.arabjc.2022.104312

[B59] Zhan P. Wang F. Xia P. Zhao G. Wei M. Wei F.. (2022). Assessment of suitable cultivation region for *Panax notoginseng* under different climatic conditions using MaxEnt model and high-performance liquid chromatography in China. Ind. Crops Prod. 176, 114416. doi: 10.1016/j.indcrop.2021.114416

[B60] Zhang Y. Tang J. Ren G. Zhao K. Wang X. (2021). Global potential distribution prediction of *Xanthium italicum* based on Maxent model. Sci. Rep. 11, 16545. doi: 10.1038/s41598-021-96041-z 34400696 PMC8368065

[B61] Zhang W. Xu H. Li C. Han B. Zhang Y. (2024). Exploring Chinese herbal medicine for ischemic stroke: insights into microglia and signaling pathways. Front. Pharmacol. 15. doi: 10.3389/fphar.2024.1333006 PMC10838993 38318134

[B62] Zhao H. Zeng S. Chen L. Sun Q. Liu M. Yang H.. (2021). Updated pharmacological effects of *Lonicerae japonicae* flos, with a focus on its potential efficacy on coronavirus disease–2019 (COVID-19). Curr. Opin. Pharmacol. 60, 200–207. doi: 10.1016/j.coph.2021.07.019 34461565 PMC8402937

[B63] Zheng S. Liu S. Hou A. Wang S. Na Y. Hu J.. (2022a). Systematic review of *Lonicerae Japonicae* Flos: A significant food and traditional Chinese medicine. Front. Pharmacol. 13, 1013992. doi: 10.3389/fphar.2022.1013992 36339557 PMC9626961

[B64] Zheng T. Sun J. q. Shi X. j. Liu D. l. Sun B. y. Deng Y.. (2022b). Evaluation of climate factors affecting the quality of red huajiao (*Zanthoxylum bungeanum* maxim.) based on UPLC-MS/MS and MaxEnt model. Food Chem.: X 16, 100522. doi: 10.1016/j.fochx.2022.100522 36519100 PMC9743291

