# Supplementary material for: Evaluating the impact of ecological factors on the quality and habitat distribution of Lonicera japonica Flos using HPLC and the MaxEnt model
Source: Front Plant Sci. 2024 Aug 6;15:1397939. doi: 10.3389/fpls.2024.1397939 (PMC11333331; doi:10.3389/fpls.2024.1397939)
Supplement: Supplementary file 1 [file DataSheet_1.docx]

Supplementary Material

# Supplementary Figures and Tables

For more information on Supplementary Material and for details on the different file types accepted, please see [here](https://www.frontiersin.org/guidelines/author-guidelines#supplementary-material).

## Supplementary Figures


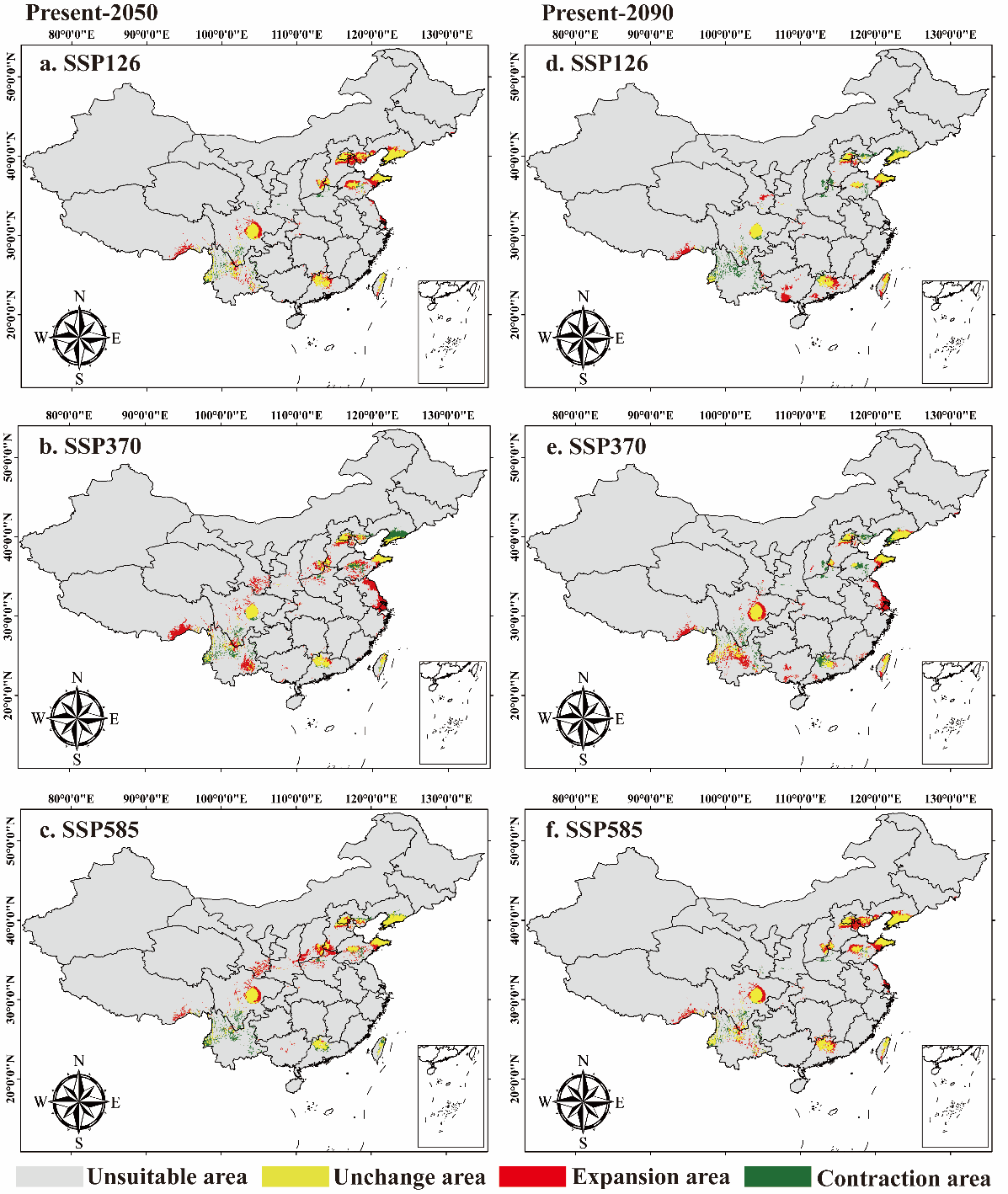


**Figure S1.** Spatial changes of LJF in China under emission scenarios of the 2050s and 2090s.


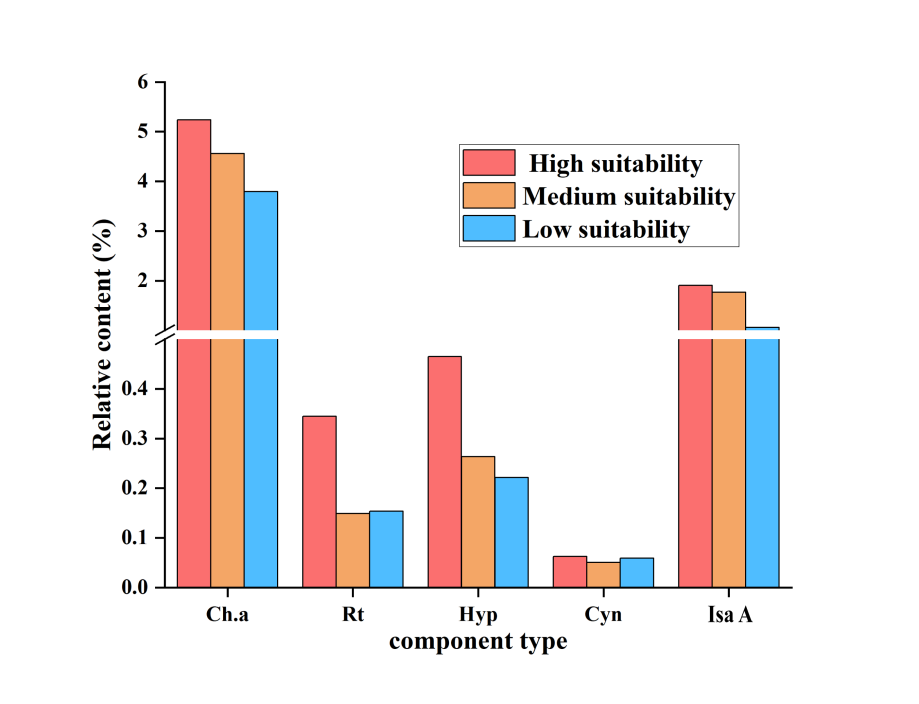


**Figure S2.** The components content (mean) map of LJF under different suitability areas.

## Supplementary Tables

Table S1. Percent contribution of each environmental variable

|  | **Period** | **Bio6** | **Bio12** | **Bio4** | **Srad4** | **Elev** | **Srad2** | **S-oc** |
| --- | --- | --- | --- | --- | --- | --- | --- | --- |
| Percent contribution | Current | 44.6 | 40.3 | 8.5 | 3.2 | 2.4 | 0.8 | 0.1 |
| Permutation importance |  | 58.1 | 25.5 | 4.6 | 1.5 | 7.7 | 2.2 | 0.4 |

Table S2 The potential distribution area of the LJF in the 2050s and 2090s.

| **Period** | | **Area of each suitable region (× 10^4^ Km^2^)** | | | | | | | |
| --- | --- | --- | --- | --- | --- | --- | --- | --- | --- |
|  |  | **Unsuitable region** | | **Unchanged region** | | **Expansion region** | | **Contractionregion** | |
| Current **vs** SSP126-2050s | | 919.96 | | 12.67 | | 6.64 | | 9.63 | |
| Current **vs** SSP370-2050s | | 905.53 | | 13.96 | | 21.07 | | 8.33 | |
| Current **vs** SSP585-2050s | | 914.04 | | 15.08 | | 12.55 | | 7.22 | |
| Current **vs** SSP126-2090s | | 916.43 | | 13.07 | | 10.16 | | 9.22 | |
| Current **vs** SSP370-2090s | | 909.80 | | 14.44 | | 16.80 | | 7.86 | |
| Current **vs** SSP585-2090s | | 911.27 | | 19.04 | | 15.32 | | 3.25 | |
